# An Incentive-Based Framework for Analyzing the Alignment of Institutional Interventions in the Public Primary Healthcare Sector: The Portuguese Case

**DOI:** 10.3390/healthcare9070904

**Published:** 2021-07-16

**Authors:** Miguel Alves Pereira, Rui Cunha Marques, Diogo Cunha Ferreira

**Affiliations:** 1 Centro de Estudos de Gestão do Instituto Superior Técnico (CEG-IST), Instituto Superior Técnico, Universidade de Lisboa, 1049-001 Lisbon, Portugal; 2 Civil Engineering Research and Innovation for Sustainability (CERIS), Instituto Superior Técnico, Universidade de Lisboa, 1049-001 Lisbon, Portugal; rui.marques@tecnico.ulisboa.pt (R.C.M.); diogo.cunha.ferreira@tecnico.ulisboa.pt (D.C.F.)

**Keywords:** primary healthcare, reform, institutional interventions, incentives, SWOT analysis

## Abstract

Over the years, the Portuguese National Health Service has undergone several reforms to face the challenges posed by internal and external factors on the access to and quality of its health services. One of its most recent reforms addressed the primary healthcare sector, where understanding the incentives behind the actors of the inherent institutional interventions and how they are aligned with the governing health policies is paramount for reformative success. With the purpose of acknowledging the alignment of the primary healthcare sector’s institutional interventions from an incentive-based perspective, we propose a framework resting on a SWOT (Strengths, Weaknesses, Opportunities, and Threats) analysis, which was built in cooperation with a panel of decision-making actors from the Portuguese Ministry of Health. In the end, we derive possible policy implications and strategies. This holistic approach highlighted the positive impact of the primary healthcare reform in the upgrade of physical resources and human capital but stressed the geosocial asymmetries and the lack of intra- and inter-sectorial coordination. The proposed framework serves also as a guideline for future primary healthcare reforms, both national- and internationally.

## 1. Introduction

The Portuguese National Health Service (SNS, from the Portuguese abbreviation of *Serviço Nacional de Saúde*) was born in 1979 as a solution to ensure equitable access to primary healthcare, secondary healthcare, and tertiary healthcare—the organization of Portugal’s levels of care, from more general to more specialized health problems—in the country, half a decade after the end of a forty-one-year-old uninterrupted dictatorship [1]. Based on a Beveridgian philosophy, the SNS delivers health services to a population of more than ten million people, governed by a Central Government, via the Ministry of Health, which aims for delivering universal, equitable, and progressively free care to its users through a tax-financed system.

Over the years, the SNS has evolved tremendously in terms of efficiency, access, quality, and sustainability [2], which placed Portugal as the thirteenth best European health system in 2018, according to the Euro Health Consumer Index (EHCI) [3]. However, studies show that Portugal, despite being a middle-income country, has followed the trend of the so-called developed countries in the sense that the population is living longer and has a lower replacement rate [4], i.e., the number of births in the population is not being able to offset the number of deaths in the long run in order for the population to remain constant. Therefore, the country is tackled by new problems in the shape of chronic diseases and unbalanced lifestyles. Consequently, according to the Organization for Economic Co-operation and Development (OECD), the health expenditure as a share of the gross domestic product has been increasing in the last five years, following a reduction in the years post-financial crisis. This places Portugal four places below the top 10 of the OECD countries regarding this indicator.

In light of these facts, the Portuguese government had to act in order to conduct a handful of reforms [5], especially in primary and secondary healthcare. Regarding the latter, since public hospitals are responsible for consuming more than half of the available resources [6], the implementation of health reforms has sought to minimize costs and maximize value for money. These reforms include corporatization [7,8], vertical and horizontal mergers of providers [6], and public–private partnerships [9,10,11], having been fairly discussed in the literature.

Regarding the former, Portugal’s primary healthcare sector is being formally delivered to the population for almost 40 years. As gatekeeper for the provision of healthcare in the country, this sector assumes a critical role in the SNS, which is the reason why it has adapted and evolved significantly over time [12] under several primary healthcare reforms. In 2008, the conditions to construct a new comprehensive model of primary healthcare management were created—the Groupings of Healthcare Centers (ACeSs, from the Portuguese abbreviation of *Agrupamentos de Centros de Saúde*)—which materialized in 2009. The ACeSs group includes several healthcare centers, whose functional units include, at least, one Family Health Unit (USF, from the Portuguese abbreviation of *Unidade de Saúde Familiar*), among other types of specific healthcare units concerning personalized care, shared care resources, public health, and long-term care.

**Figure 1:**
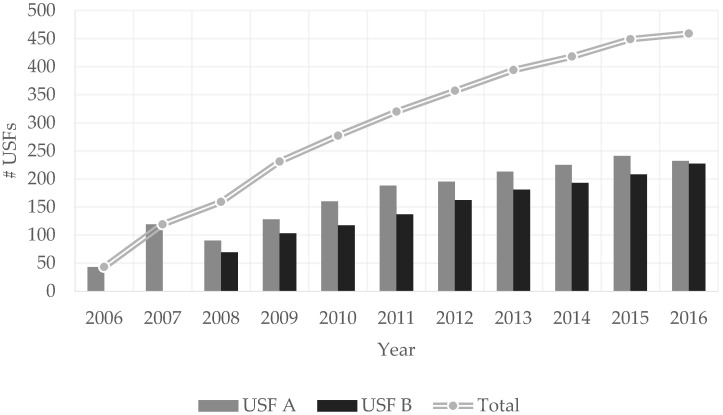
Evolution of the total number of USFs (2006–2016). Source: ACSS [14].

At the moment, this reform, initiated in 2005, has been targeting the reinforcement and the expansion and reorganization of the primary healthcare network through the deepening of the model and the performance of its functional units, especially USFs, with the purpose of increasing the SNS’s responsiveness and assigning a USF team to every resident in Portugal [13]. Currently, the number of USFs has almost multiplied tenfold since 2006, as we can see in Figure 1, based on the only available data provided by the Portuguese Central Administration of the Health System (ACSS, from the Portuguese abbreviation of *Administração Central do Sistema de Saúde*) [14]. Note that USFs can evolve according to three models, which will be detailed in Section 2.

Future primary healthcare reforms plan on adding public health measures, e.g., embed dental medicine in the SNS, implementing countrywide cancer screening projects, providing complementary means of diagnostic to the primary healthcare’s functional units, and further developing mobile healthcare units. Some authors have pointed out that governance mechanisms do facilitate improvements in public health, e.g., malaria control [15].

However, it is universally recognized by the international community the emphasis on the need to understand local contexts by considering their specific constraints and alternatives in a “no-one-size-fits-all” solution, which is the basis of New Public Governance (NPG) [16]. Undeniably, combining robust frameworks with legal and regulatory “best practices” proved not to benefit institutions, which in turn required a deeper knowledge of the political foundations, development drivers, decision-making processes, and interactions between de jure and de facto institutions. Consequently, acknowledging the incentives of the actors of an institutional reform is fundamental to move it forward in a dynamic, committed, and accountable fashion [17].

For this reason, we propose a framework, inspired by the works of Mumssen, Saltiel, and Kingdom [17] and Tavares [18], that intends to understand how the Portuguese 2005 primary healthcare reform, which is still underway, is aligned in terms of policies, institutions, and regulations, focusing on their incentives aimed at achieving potentially significant financial and social changes via a SWOT (Strengths, Weaknesses, Opportunities, and Threats) analysis, constructed alongside decision-making actors of the Portuguese Ministry of Health. Ultimately, this work serves the purpose of providing the foundations to aid actors involved in wider health policy making to understand how the enabling environment that surrounds them and the drivers that steer the aforementioned institutional interventions of their own primary healthcare reforms can lead to a more sustainable future.

## 2. Methods

Solo policy, institutional, or regulatory interventions are not enough. It is in the appropriate mix of the three institutional interventions that the alignment of the incentive structures is based on [19,20]. Aligning reformative drivers with the incentives created through institutional interventions is the key to developing sustainable incentive structures. Thus, a holistic framework needs to look at the alignment of policies, institutions, and regulations of the Portuguese primary healthcare as a whole and provide a structured view of the sector.

One way to do this is by means of a SWOT analysis. Despite being an established approach to structured planning, SWOT analysis finds its only application in health policy in Portugal in the work of Tavares [18], concerning the Portuguese health system, where the author uses SWOT tools to structure a literature review to better understand the situation of the SNS. Our study differs from the one of Tavares [18], since it concerns the primary healthcare sector and incorporates an expert panel, with a view to possible decision aiding. An application of this tool in primary healthcare can be found in, e.g., van Durme et al. [21], where the authors attempted to perceive the stakeholders’ standpoint on the organization of chronic care in Belgium.

By adapting the four traditional steps of a SWOT analysis for strategic planning in healthcare, as proposed by Harrison [22], we were able to establish our framework by combining the reformative drivers and the enabling environment of the primary healthcare reform and infer its strengths, weaknesses, opportunities, and threats with the assistance of a group of decision-making actors from the Portuguese Ministry of Health. Therefore, our framework consists of the following:

1. Collecting information on the reformative drivers and the enabling environment of the Portuguese primary healthcare sector reform;
2. Organize the collected information according to SWOT categories;
3. Develop a SWOT analysis with the group of decision-making actors;
4. Use the results of the SWOT analysis to derive policy implications.

Section 3.1 contains the first step of the framework, while Section 3.2 further describes the features of the remaining steps and the results. Section 4 presents the discussion.

## 3. Results

### 3.1. Reformative Drivers and the Enabling Environment

In the face of the socio-economic scenario portrayed in Section 1, the Portuguese government and the SNS have been trying to adapt and respond to the new needs of progressively more informed and demanding citizens. Nonetheless, from theory to practice goes a long way, given the number of assorted factors that influence primary healthcare interventions at policy, institutional, and regulatory levels.

One of the keys to these issues lies in incentives. Incentives are the result of sound institutional interventions and stimulate the actors of the primary healthcare services to pursue a set of objectives and to behave in a certain way [17]. Such actors may be players, stakeholders, and decision-makers from regulatory agencies and national and local governments, not to mention the users and the staff of the services, their associations, and even unions.

According to Mumssen, Saltiel, and Kingdom [17], there are two broad types of incentives: those that are derived from the enabling environment and those that are derived from policies, institutions, and regulations. The drivers that initiate and carry out a reform in the primary healthcare sector are influenced by the status quo of the public sector and general government policies, and they can come from endogenous (e.g., international agreements and national initiatives) or exogenous sources (e.g., external frameworks imposed by international agencies).

In essence, aligning exogenous and endogenous drivers is crucial for the development of appropriate institutional interventions [17], which is also true regarding the SNS’s primary healthcare services. Starting with raising the awareness of external and non-decision-making internal actors to the intricacy of the Portuguese healthcare context, the chances that they will reinforce endogenous reformative drivers and work more closely with the government are higher. For instance, studies of groups of health professionals have established non-formal reform measures of performance-based incentives throughout the years in primary healthcare [23]. This is supported by Oliver [24] in his performance-management-driven incentives.

#### 3.1.1. Policies and Incentives

Policies, especially public ones, are exceedingly flexible concepts that, nonetheless, can be defined as frameworks that guide governmental decision making toward taking certain actions and achieving specific goals. For instance, they can be implemented as laws or regulatory measures and ultimately develop accountability between a government and its citizens [25]. A policy is more effective if it fits the specific local political economy and governance context [26].

Therefore, although the same can happen in the opposite situation, the lack of coherent and consistent policy objectives and targets leads to the failure in delivering the envisioned incentives [26]. This is what happened by the time the third generation of primary healthcare services was supposed to have been operationalized in the early 1990s [27]. Since the government did not create straightforward policies through sustainability-promoting strategies, the primary healthcare reform was adrift for almost a decade. Yet, nowadays, in what can be labeled as the “fourth generation” of that reform, the restructuring of the network of primary healthcare is well underway, as explained in Section 1.

Moreover, the disequilibrium between primary healthcare and secondary healthcare human resources was tremendous, with the higher preference of clinical staff for working in the latter’s services. One of the policy interventions of the primary healthcare reform proposes measures that incentivize healthcare professionals to opt for the provision of primary healthcare, in order to reduce that deficit [19]. Still, only with adequate payment mechanisms can primary healthcare services attract skilled human capital, such as the incentive-based approach for mental health primary care practices proposed by Perelman et al. [28]. Note that the clinical practice guidelines are also influenced by financial incentives, among other factors (e.g., training, regulation, and institutional support), which constitute one of the main barriers to their implementation [29].

Another effort of the Ministry of Health is to bring primary healthcare systems to the present. Developing a suitable information system not only tackles the deficiencies of current practices but also promotes a better quality of care [27]. The adoption of such technologies by the actors in primary healthcare services is highly incentivized with the help of professional development training sessions and state-of-the-art equipment [30,31].

Achieving accreditation is also a possible incentive for primary healthcare services to improve their quality [32], but there are no items on the governmental agenda in that sense so far.

#### 3.1.2. Institutions and Incentives

Institutions are social, political, and economic relations overseen by formal and informal rules. They represent the structures through which people interact and incentives are shaped, and they outline the roles of its actors. Depending on the type of relations and actions they promote, institutions can generate positive or negative incentives [33].

The reconfiguration of the ACeSs and their autonomy is an example of an institutional intervention conducted by the primary healthcare reform. In this new model, the possibility to influence the guidelines of their respective ACeS is up to the citizens, which is an innovative measure that places Portugal in the lead among developed countries. Community participation has become increasingly more prominent in the global dialogue surrounding primary healthcare reforms [34].

Accomplishing the implementation of the USFs and their production-based incentives [35] is another positive formal institutional intervention. Furthermore, some authors claim that reorganizing traditional primary healthcare units into USFs might result in more efficient services and higher access to healthcare [36]. Indeed, the possibility to evolve within the USF framework is foreseen in the law, since growing from the so-called ‘Model A’ to ‘Model B’ and from ‘Model B’ to ‘Model C’ will be accompanied with the corresponding incentives [37]. In particular, USFs A correspond to a learning phase, comprehending public sector USFs and granting them the possibility of additional services and goals that are translated in terms of institutional incentives that can then be invested in those USFs; USFs B are indicated for organizationally mature teams, with special incentives for clinical staff and institutional incentives for as in Model A USFs; USFs C are experimental models with a supplementary nature regarding possible shortcomings of the SNS. Note that, at the moment, there are only Model A and Model B USFs, as seen in the evolution portrayed in Figure 1.

The restructuring of public health services gives a boost to the sector by strengthening its functional units with instruments that allow their actors to worry less about other issues and to focus more on increasing the effectiveness of their services to create a safer environment for the Portuguese citizens [30].

Community intervention is another dimension of the primary healthcare reform. In addition to the creation of specific functional units to provide psychological support and social healthcare services to those in need, a program of development of continued care was designed to target particularly vulnerable or isolated social groups [19,38], which is something that is already a reality in other countries, such as some rural areas of the United States of America [39].

The decentralization of primary healthcare services, driven by efficiency improvements, is something that has been occurring for a few years. In the interest of responding to the increasing demand and complexity, ACeSs were divided into functional units to provide expert services in a more coordinated and competent way.

Additionally, an incentive package to integrate healthcare both at a primary healthcare and a secondary healthcare level was designed to add value to the SNS’s users. This program implies a certain degree of articulation between healthcare institutions in what regards, e.g., early diagnosis programs; a reduction in avoidable hospitalizations, medical appointments and medical emergencies; and domiciliary support. Models relying on team interdisciplinarity to coordinate patient care have already been proposed by Korda and Eldridge [40].

Finally, given the dearth of motivation and hard and soft skills of many actors of the primary healthcare services, institutional incentives were fashioned to ensure that healthcare professionals and other staff members embraced the new organizational model of primary healthcare delivery and had at their disposal suitable working conditions and training sessions [19].

#### 3.1.3. Regulations and Incentives

Regulations can be seen as the set of rules that are created to manage a system by enforcing primary legislation. However, regulatory interventions must be intimately aligned with their context of application [17]. Hence, the effectiveness of regulatory incentives depends on regulatory goals, the framework and the capacity of the institutions to which they will be applied, the availability of information, and the strength of the political support [41].

The Health Regulatory Entity (ERS, from the Portuguese abbreviation of *Entidade Reguladora da Saúde*) is the Portuguese health regulator. As an independent public entity responsible for regulating the activity of the healthcare providers’ institutions, its sphere of influence includes all of the mainland’s public, private, and social facilities. Still, the urgency to create a structure in charge of designing, implementing, and evaluating primary healthcare reforms was already stressed out by Pisco [41], bearing in mind the balance between outputs (i.e., measures of quality and access) and outcomes (i.e., consequences of a number of complementary outputs) as key performance indicators.

If, in secondary healthcare, the majority of problems that require ERS intervention are usually related to constraints in the access to emergency services, the typical complications at a primary healthcare level are associated with constraints in the access to the scheduled provision of healthcare in ACeSs. As shown by ERS [42], 32% of the citizens were dissatisfied with the waiting time at their local ACeS, not to mention that 46% of them had to wait for their appointment for over a week since scheduling it. Interestingly, when compared with other Portuguese public services, primary healthcare services had 94% of positive opinions, which suggests an adequate level of satisfaction. Indeed, pay-for-performance incentives based on the quality of care and patient satisfaction would be a suitable solution, as suggested by Robinson et al. [43].

Nevertheless, ERS still notes that there are some regional asymmetries in the North of the mainland, viz., Vila Real, Bragança, and Viseu. It is in these areas that institutional interventions need to focus more in depth.

### 3.2. SWOT Analysis

Using the information collected in Section 3.1 alongside a descriptive and non-quantitative method—the SWOT analysis—we can continue following the steps of our framework to analyze primary healthcare strategies from the particular perspective of institutional interventions and derive policy implications. This strategic planning tool enables the identification of the positive and negative aspects of an entity or a system to analyze it and propose appropriate strategies. On the one hand, opportunities and threats are regarded as consequences of external influences; on the other hand, strengths and weaknesses are seen as internal features that can be compared with those of other entities or systems.

Built cooperatively in two meetings similar to decision conferences with a group of three decision-making actors from the Portuguese Ministry of Health specialized in health policy making and health administration, we acted as analyst/facilitators, mediating an agreement between the parts, analogously to the procedure used by Pereira et al. [44]. In essence, the cooperative SWOT analysis allowed us to build a strategy to consolidate the institutional interventions of primary healthcare services based on the incentives described in Section 2 and reflect on options for future research [35]. Table 1 contains the results of the application of our framework, which was based on research conducted in 2019.

#### 3.2.1. Strengths

By looking at the strengths of the institutional interventions of the primary healthcare sector, the actors realized that the reorganization into ACeSs was uplifting, because it was able to modernize the delivery of primary healthcare by updating the past, combining technology and a network organizational structure, and, ultimately, improving the satisfaction of the users. This was also emphasized by the creation of several functional units, with the USFs being the main entities, which complemented the provision of distinct healthcare services in this decentralized model.

Furthermore, the increased responsibility of the clinical governance of each functional unit leads to improved results that find their justification in the greater number of model B USFs. Bear in mind that the skilled and incentivized human capital assigned to the primary healthcare sector delivers a higher quality of care and improved the overall users’ satisfaction. In addition, the creation of new state-of-the-art functional units within ACeSs and the progressive evolution of USFs from Model A to Model B is a favorable point that denotes the success of the primary healthcare reform’s institutional incentives.

#### 3.2.2. Weaknesses

However, the decision-making actors identified a few weaknesses in the institutional interventions of the primary healthcare reform. First, despite the incentives toward the procurement of clinical staff, there is still a shortage of health professionals in the primary healthcare sector, especially general practitioners. This limits the access to care and, consequently, the efficiency of the service. Second, the advantages of a multidisciplinary structure can also be its disadvantages, since the lack of coordination between some health units hinders the appropriate functioning of the primary healthcare sector. Consequently, inefficiencies arise, which can also be due to the deficient timeliness of services.

#### 3.2.3. Opportunities

Two situations that pose themselves as opportunities are the progressive decentralization in the sector and the creation of partnerships with local governments, which have a great impact on the autonomy of primary healthcare services and the sustainability of the SNS, respectively. Additionally, the continuation of the current governmental primary healthcare reform is on the right path to carry on upgrading the services provided by the sector, given its modern and innovative nature. In addition, governmental initiatives to revitalize the interior regions of Portugal, resting on a more adequate resource allocation, will certainly improve the access to primary healthcare services.

#### 3.2.4. Threats

At last, the threats to the primary healthcare reform can be found in several aspects. First, although ACeSs are supported by legislation that grants them administrative autonomy in a way that makes them decentralized from their respective Regional Health Administrations (ARSs, from the Portuguese abbreviation of *Administrações Regionais de Saúde*), there are still issues that limit the autonomy of their primary healthcare units, which also affects the efficiency of the service. Additionally, despite belonging to the same ARSs, public hospitals and ACeSs experience some further lack of coordination. This results in difficulties in the access to emergency services and the scheduling of medical appointments in a reasonable time.

Second, the increasing debt of the SNS, particularly due to the expenditure of public hospitals, may take its toll in the future of the reform and the maintenance of the institutional incentives. Moreover, the better performance of Bismarckian health systems (see the EHCI ranking) in the past few years may have an impact on the structure of the SNS and, consequently, the primary healthcare sector and the alignment of its institutional interventions in the medium term, which would influence the satisfaction of the SNS‘s users and the goal of reducing geographical and social asymmetries.

Third, the geographical and social asymmetries already highlighted by the ERS still hinder the access to primary healthcare by the Portuguese population. In fact, the growing number of USFs does not yet tackle the problems caused by inland desertification, whereas the payment of user charges, even if relatively low, continues to pose an obstacle to the one-fifth of the population that reportedly lives in a condition of poverty. Otherwise, hospitalization rates will tend to increase [45]. From another angle, as in other countries in a similar socioeconomic situation as Portugal, the increasingly aging population and the absence of a culture that celebrates aging and respects the elderly has been leading to an overflow of primary healthcare facilities. This prevents an adequate delivery of health services to the remaining population strata.

## 4. Discussion

After analyzing the incentive-based institutional interventions of the Portuguese primary healthcare sector by resorting to the proposed framework, some strategies can be suggested based on the pairwise relationships of the different SWOT components. Regarding strengths/opportunities strategies, the role played by ACeSs is vital for the progressive decentralization of the primary healthcare sector, given not only the legal nature of the entities themselves but also the possibility granted by the existence of its functional units in establishing partnerships with local governments in terms of adding public health measures, such as education, dental medicine, screening platforms for cancer and chronic diseases, mobile healthcare units, and complementary means of diagnostic [19]. As a matter of fact, these partnerships may prove their usefulness in reducing the existing geosocial asymmetries.

In addition, in what concerns weaknesses/opportunities strategies, central and local government initiatives can constitute strengths in the sense that they may be able to provide specific incentives for dealing with the lack of health professionals in the primary healthcare sector; in particular, creating the conditions to maintain the qualified healthcare professionals with degrees produced by Portugal is a challenge already emphasized in the literature, especially nurses [18]. This would have an impact on the quality of service, which would not only reduce waiting times but also increase user and personnel satisfaction. Additionally, despite the major investment in state-of-the-art facilities, further investments in a unified information system would significantly improve the discoordination between functional units and the digitalization of health, leading to a new age of person-focused medicine in line with the current concerns surrounding mental health and chronic diseases [19].

Moreover, strengths/threats strategies necessarily imply leveraging the autonomy of ACeSs and its multidisciplinary functional units to minimize the inefficiency of the system as a whole, given their independence to increase the alignment with their local secondary healthcare providers, as well as minimize the geosocial asymmetries, due to the implementation of local programs suitable for the communities. Furthermore, internal incentives aimed at the human capital of each functional unit reduce the need for additional governmental funds to supplement possible service gaps, since the staff is as skilled as it is motivated [27].

Finally, weaknesses/threats strategies must revolve around countering the shortage of health professionals (with further incentives aimed at the clinical staff), the discoordination between units and sectors (with the adoption of a unified information system), and the geographical and social asymmetries (with initiatives derived from local ACeSs and partnerships with local governments). Regulatory interventions could also be crucial to ensure that incentives, policies, and systems are well implemented, with the ERS having to assume a key part in the strategic plan. In fact, promoting NPG’s principles of accountability and transparency could improve the system’s performance, as seen in other sectors [46].

## 5. Conclusions

In essence, the proposed framework showed the gaps between the desires of policy-makers and the reality faced by healthcare professionals and the users of the SNS. With this in mind, it is clear that the implementation of institutional interventions must be accompanied by the appropriate allocation of resources [37] and fitting governance rules [47]. This would strengthen primary healthcare services, reduce the frictions between actors and entities, and, ultimately, serve the needs of the population. For all intents and purposes, this exercise proved that the use of simple rational tools produces a better understanding of the existing alignment of institutional interventions, which, in turn, builds the structure for the planning of adequate responses.

In conclusion, since the purpose of this work was to present a framework for looking at the current alignment of the Portuguese primary healthcare sector’s institutional interventions from an incentives’ standpoint, we can safely state that a concise contemporary view on the subject was achieved to guide future research, further primary healthcare reforms, and aid key actors of policy, institutional, and regulatory interventions. Still, our analysis contains some limitations, since, similar to other classical SWOT analyses, it merely identifies issues and does not provide solutions (only suggestions to address those issues). In addition, although it is based on an extensive literature review on the reformative drivers and the enabling environment of the Portuguese primary healthcare sector and is constructed with the aid of a panel of experts, it may oversimplify or even overlook information and possible trade-offs (for instance, the current COVID-19 pandemic and its impacts were not considered).

Despite the limitations, the study of the alignment of institutional interventions of secondary healthcare, the relationship between primary healthcare and secondary healthcare, and a comparison between health systems of distinct countries would be interesting to follow, as well as on the assessment of the performance impacts that the incentives behind the primary healthcare institutional interventions have had (something we were unable to do here due to lack of data).

## Figures and Tables

**Table 1:** SWOT analysis of the alignment of institutional interventions on the Portuguese primary healthcare.

**Strengths**	**Weaknesses**
The creation of the ACeSs ^1^ was vital to revitalize the primary healthcare sector.	The shortage of health professionals is still a problem.
The myriad of multidisciplinary primary healthcare functional units complements the existing primary healthcare services.	The lack of coordination between health units generates obstructions to a suitable institutional alignment.
The skilled and incentivized human capital delivers a higher quality of care.	The inadequate timeliness of services brings about tremendous inefficiency and user dissatisfaction.
The state-of-the-art primary healthcare facilities and equipment upgrade the level of care.	
**Opportunities**	**Threats**
The progressive decentralization and creation of partnerships with local governments.	The centralization of management blocks the autonomy of primary healthcare units.
	The lack of coordination between primary and secondary healthcare produces further challenges to an adequate institutional alignment.
	The increasing debt of the SNS ^2^ dangerously threatens the sustainability of health provision in Portugal, including primary healthcare services.
	The geographical and social asymmetries hinder universal access to primary healthcare.
The current governmental primary healthcare reform is a big step toward upgraded primary healthcare services.	The aging Portuguese population is overflooding primary healthcare facilities and preventing the provision of care to all strata.
The governmental initiatives to revitalize the interior regions of Portugal include the allocation of resources toward improving the access to primary healthcare services.	The better performance of Bismarckian health systems may cause the need to design a new primary healthcare reform and realign the incentives of key actors.

^1^ Groupings of Healthcare Centers (ACeSs, from the Portuguese abbreviation of *Agrupamentos de Centros de Saúde*); ^2^ Portuguese National Health Service (SNS, from the Portuguese abbreviation of *Serviço Nacional de Saúde*).

## Data Availability

No new data were created or analyzed in this study. Data sharing is not applicable to this article.
